# Supplementary material for: Mechanical Loading-Driven Tumor Suppression Is Mediated by Lrp5-Dependent and Independent Mechanisms
Source: Cancers (Basel). 2021 Jan 13;13(2):267. doi: 10.3390/cancers13020267 (PMC7828232; doi:10.3390/cancers13020267)

cancers

Supplementary materials: Mechanical Loading-Driven Tumor Suppression Is Mediated by Lrp5-Dependent and Independent Mechanisms

Yan Feng, Shengzhi Liu, Rongrong Zha, Xun Sun, Kexin Li, Alexander Robling, Baiyan Li and Hiroki Yokota

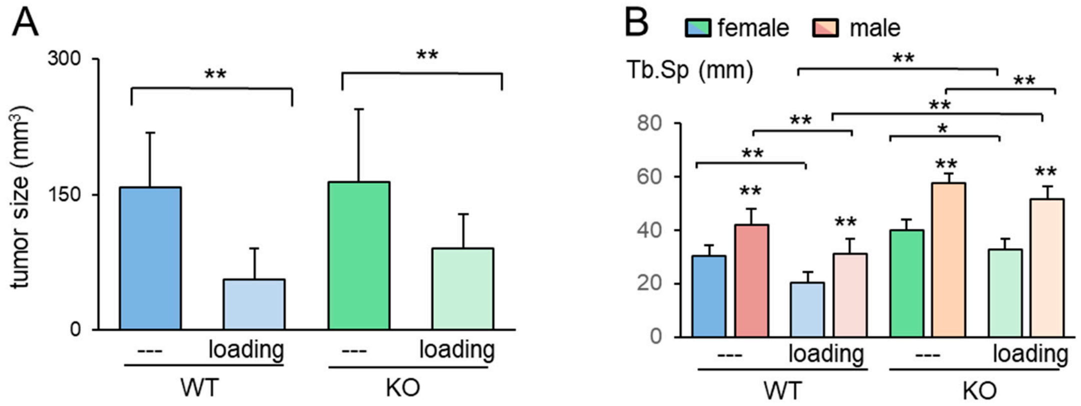

Figure S1. Loading-driven reduction in mammary tumors and the protection of tumor-invaded tibia. KO = knockout, and WT = wild-type. The single and double asterisks indicate $p < 0.05$ and 0.01, respectively. Tb.Sp = trabecular separation. (**A**) Tumor size in four groups. (**B**) Trabecular separation (Tb.Sp) based on microCT images in the proximal tibiae in the wild-type and knockout mice.

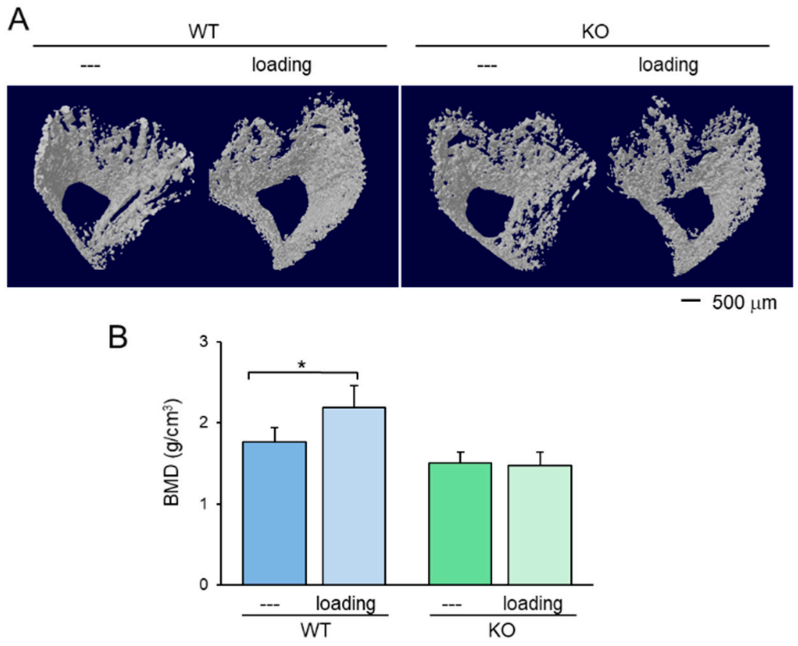

Figure S2. Effect of knee loading on the tibial cortical bone without tumor inoculation. WT = wild-type, KO = knockout. The single asterisk indicates $p < 0.05$. (**A**) micro CT images. (**B**) Bone mineral density (BMD).

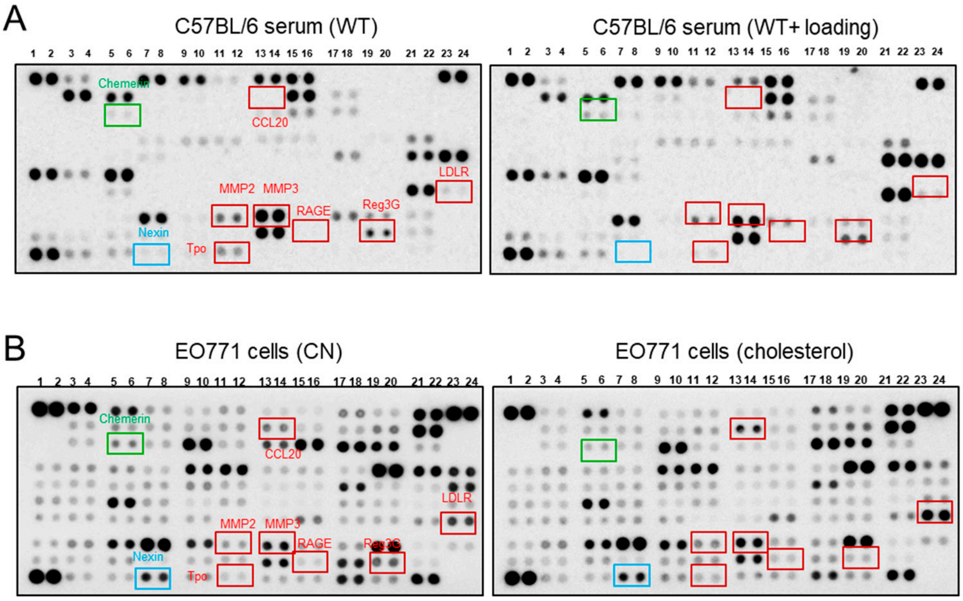

Figure S3. Cytokine array analysis using the serum and EO771 mammary tumor cells. WT = wild-type, and CN = control. (**A**) Cytokine expression profile in the serum of wild-type mice with and without knee loading. (**B**) Cytokine expression profile in EO771 cells with and without the administration of 10 μM cholesterol.

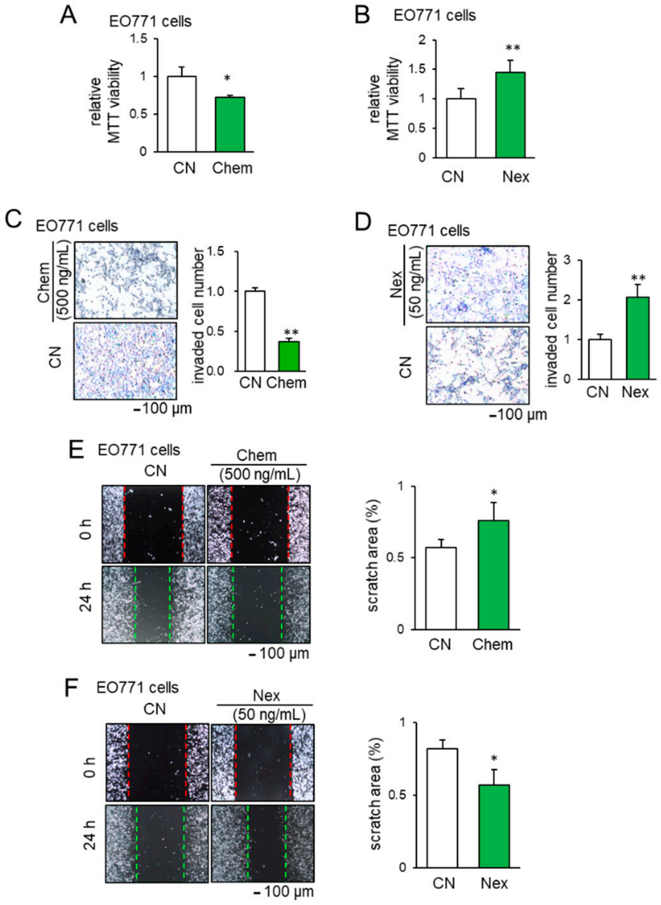

Figure S4. Responses of EO771 mammary tumor cells to chemerin and nexin. CN = control, Chem = chemerin, and Nex = nexin. The single and double asterisks indicate $p < 0.05$ and 0.01, respectively. (**A**,**B**) MTT-based viability in response to chemerin and nexin. (**C**,**D**) Transwell invasion in response to chemerin and nexin. (**E**,**F**) Scratch-based migration in response to chemerin and nexin.

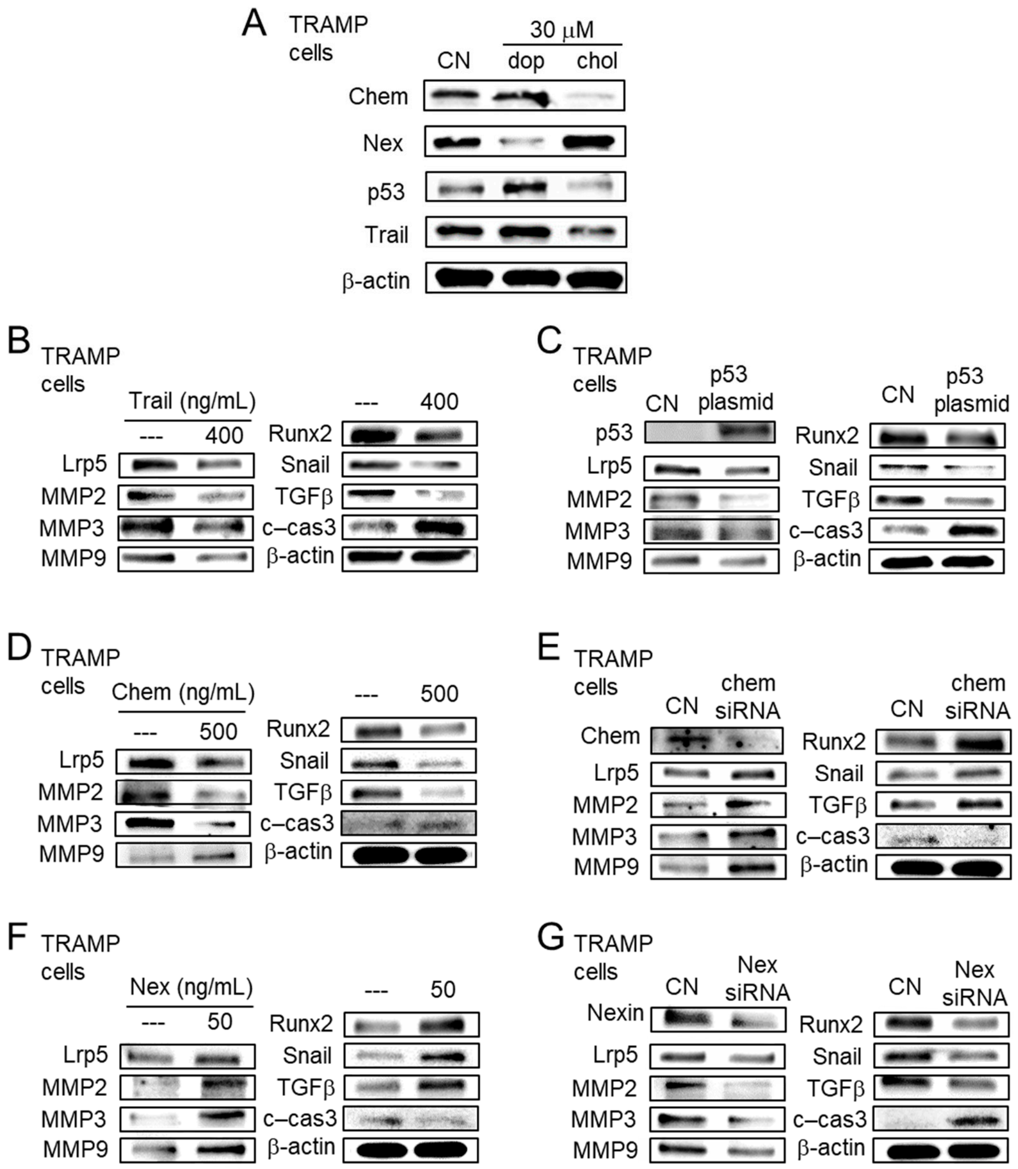

Figure S5. Expression of Lrp5, MMP2, MMP3, MMP9, Runx2, Snail, TGFβ, and cleaved caspase 3 in transgenic adenocarcinoma of the mouse prostate (TRAMP) prostate tumor cells. CN = control, c-cas 3 = cleaved caspase 3, Chem = chemerin, and Nex = nexin. (**A**) Response to dopamine and cholesterol. (**B**) Response to the administration of TRAIL. (**C**) Response to the transfection of p53. (**D**) Response to the administration of chemerin. (**E**) Response to RNA interference with chemerin siRNA. (**F**) Response to the administration of nexin. (**G**) Response to the administration of RNA interference with nexin siRNA.

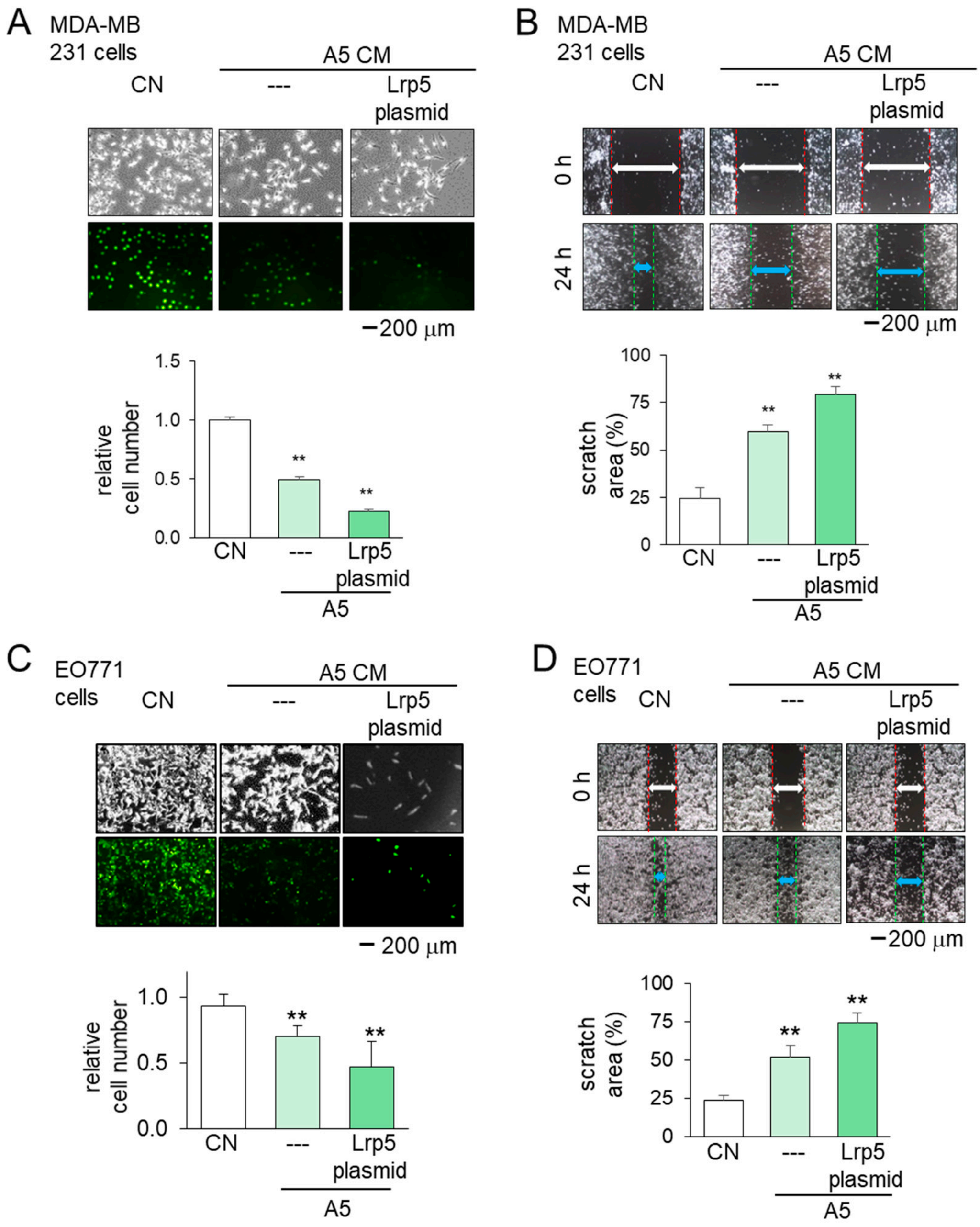

Figure S6. Effects of Lrp5-overexpressing osteocyte-derived conditioned medium on the proliferation and migration of tumor cells. CN = control, CM = conditioned medium, and A5 = MLO A5 osteocytes. (**A**,**B**) Reduction in EdU-based proliferation and scratch-based migration o MDA-MB-231 breast cancer cells by the osteocyte-derived conditioned medium with and without the overexpression of Lrp5. (**C**,**D**) Reduction in EdU-based proliferation and scratch-based migration of EO771 mammary tumor cells by the osteocyte-derived conditioned medium with and without the overexpression of Lrp5.

Original images:

Supplementary uncropped gel images for Figure 4 E.

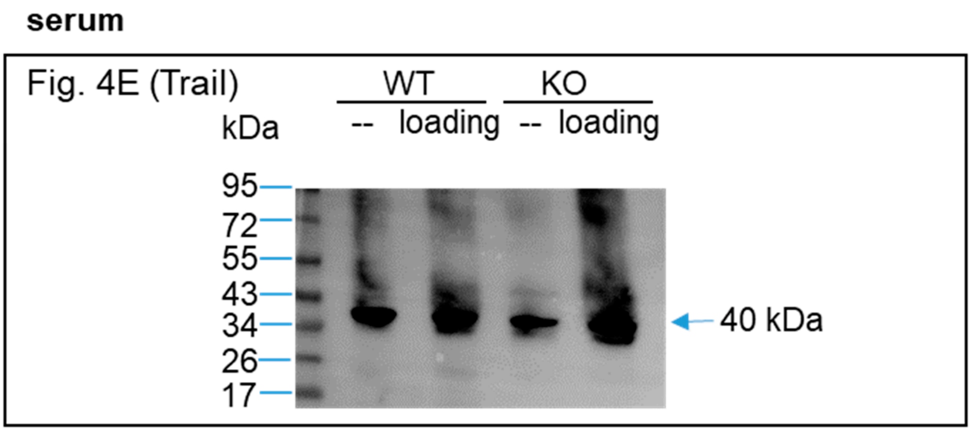

Supplementary uncropped gel images for Figure 5 A&E.

EO771 cells

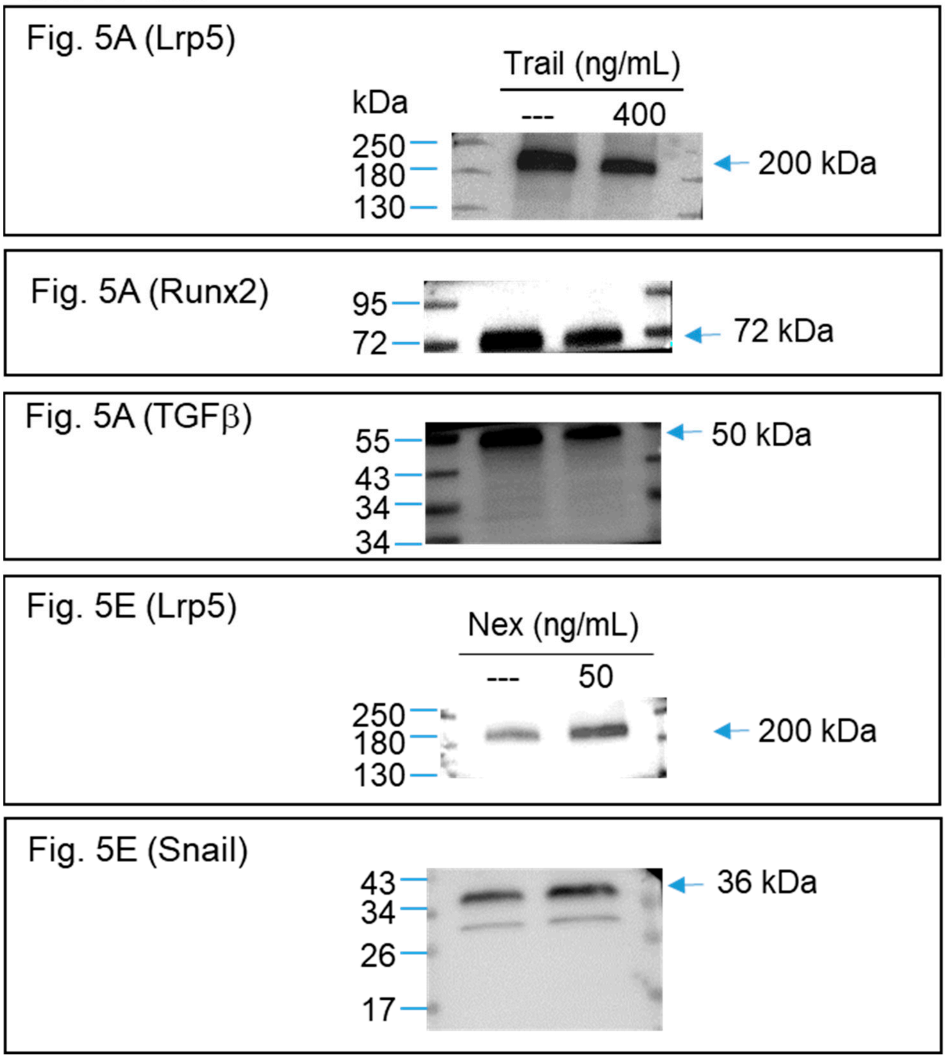

Supplementary uncropped gel images for Figure 5C.

EO771 cells

Fig. 5C (MMP2)

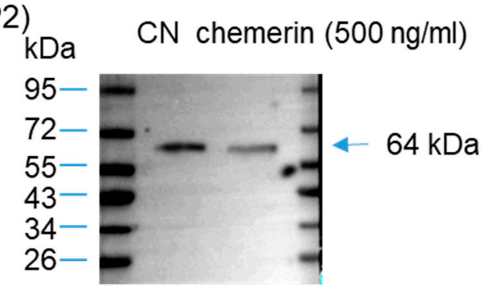

Fig. 5C (MMP3)

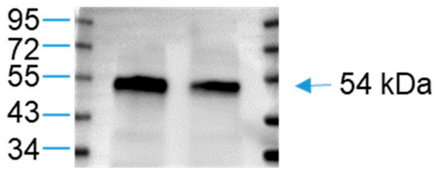

Fig. 5C (c-caspase 3)

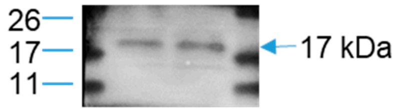

Supplementary uncropped gel images for Figure 6 B&E.

A5 osteocytes

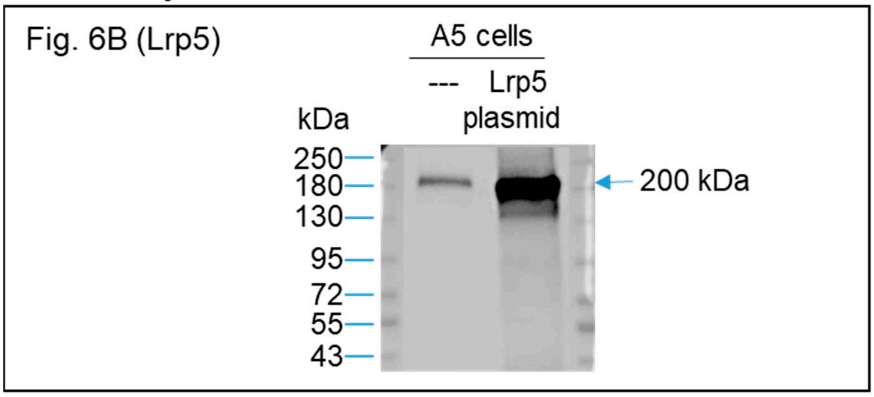

EO771 cells

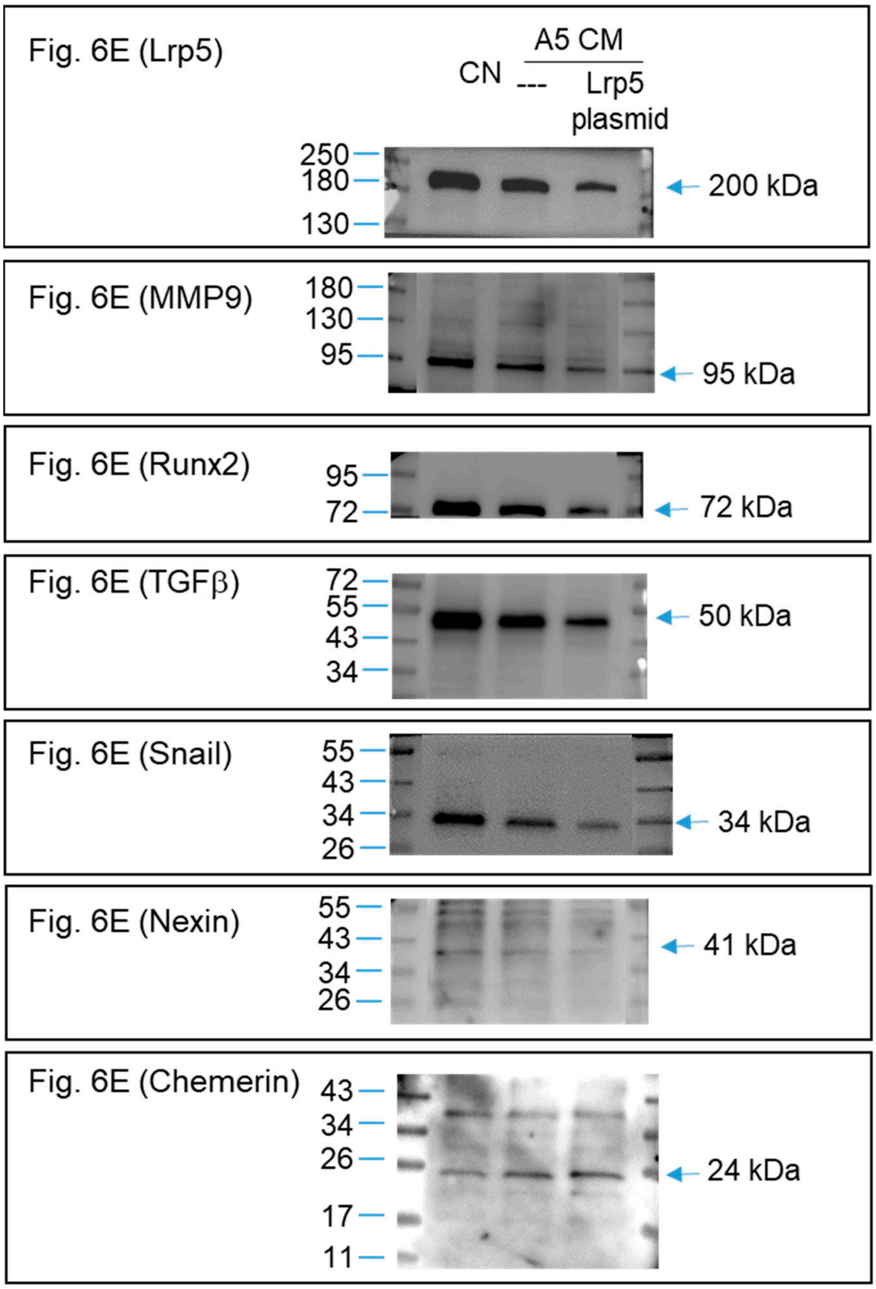

Supplementary uncropped gel images for Figure 6G&H.

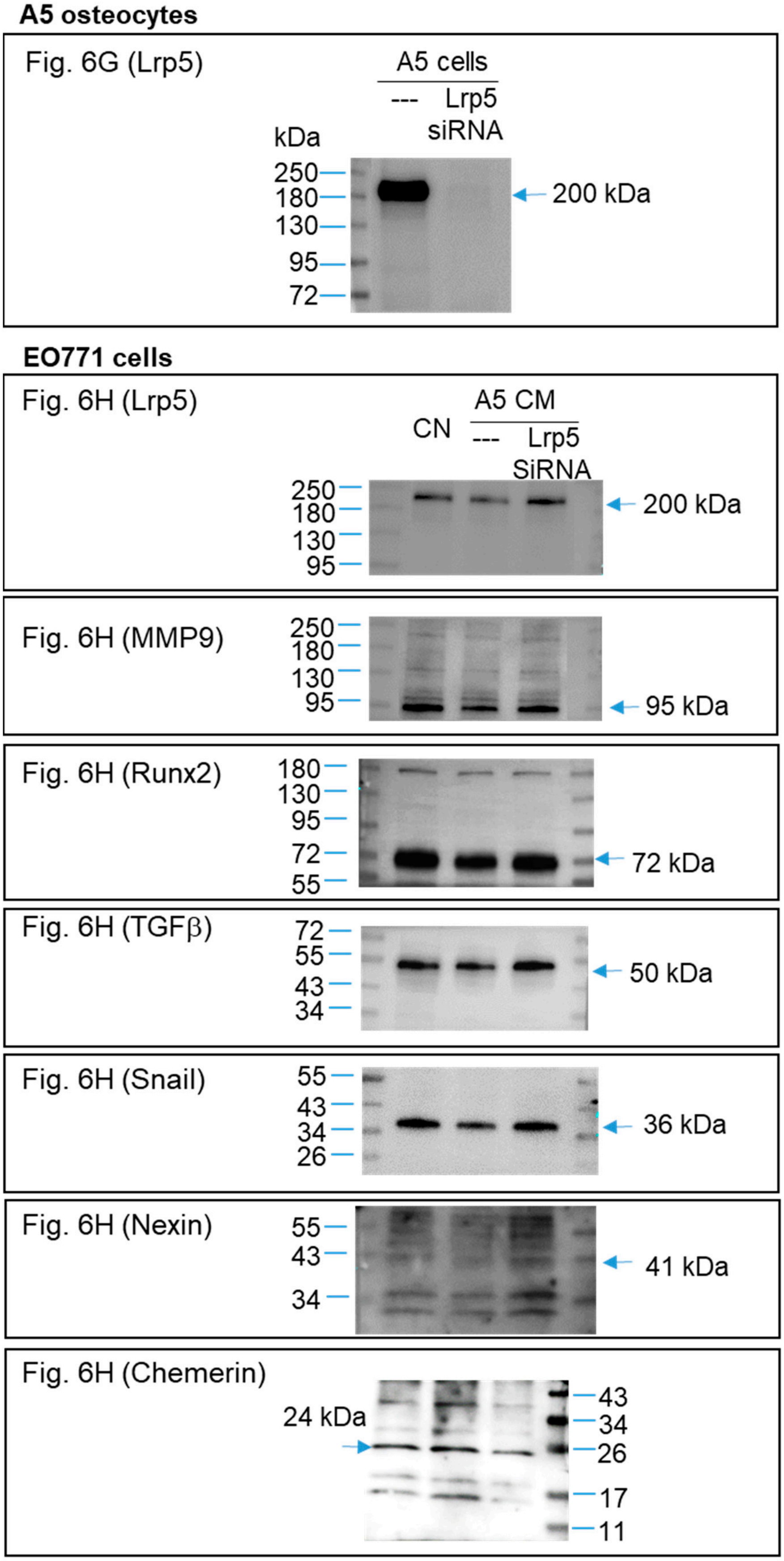

Supplement: Supplementary file 1 [file cancers-13-00267-s001.pdf]
